# Oxidation Behavior of Fe–Al, Fe–Si and Fe–Al–Si Intermetallics

**DOI:** 10.3390/ma12111748

**Published:** 2019-05-29

**Authors:** Pavel Novák, Kateřina Nová

**Affiliations:** Department of Metals and Corrosion Engineering, University of Chemistry and Technology, Prague, Technická 5, 166 28 Prague, Czech Republic; novakx@vscht.cz

**Keywords:** oxidation, iron aluminide, iron silicide, Fe–Al–Si alloy

## Abstract

Iron aluminides are still deeply investigated materials for their use in power plants, automotive and chemical industry, and other sectors. This paper shows that it is possible to strongly improve their oxidation behavior by the addition of silicon. The description of the synergic effect of aluminum and silicon on the oxidation behavior of Fe–Al–Si alloys at 800 °C in air is presented. The oxidation rate, microstructure, phase, and chemical composition of these ternary alloys are compared with the binary Fe–Al and Fe–Si alloys. Results showed that the oxidation of Fe–Al–Si ternary alloys provides an oxide layer based on aluminum oxide with a low concentration of iron and silicon. Below this oxide layer, there is a layer of silicides formed as a result of depletion by aluminum, which forms a secondary oxidation protection.

## 1. Introduction

Iron aluminides have attracted considerable attention since the 1950s due to their very good resistance against oxidation and sulfidation atmosphere [1]. In that period, the materials based on Fe–Al–C system had been developed, having been based on cast iron with the addition of aluminum [1]. These alloys were composed of B2 iron aluminide (FeAl) matrix and aluminum carbide particles [1]. The aluminum carbides can exist in two forms—sole Al_4_C_3_ carbide and mixed Fe_3_AlC carbide with perovskite structure [2,3]. In fact, the existence of Al_4_C_3_ carbide is the biggest problem with this group of materials, because it can form methane when in contact with acidic environments or water vapor at high temperatures [4]. Therefore, nowadays the iron aluminide materials without carbon are of high interest. There are numerous studies on the effect of particular alloying elements, such as Cr, Nb and Zr, on mechanical properties and high-temperature oxidation behavior [5,6,7,8]. The alloying element that attracted high attention from our team, is silicon. This element was contained already in Fe–Al–C alloys made of cast iron but in a very small amount. In these materials, it affected the type of carbide phase (Al_4_C_3_ vs. Fe_3_AlC) [2], but its effect on the properties was not studied in the latter referred paper. In 2011, our team reported that silicon could increase the oxidation resistance of Fe–Al-based alloys [9]. In fact, the paper compared the oxidation behavior of Fe–Al–Si alloys with various contents of silicon and aluminum. It was found that the silicon-rich FeAl20Si20 (in wt %) exhibited the superior oxidation resistance among the tested alloys. 

However, the oxidation behavior was affected and somewhat distorted by significant porosity, because the alloys, prepared by self-propagating high-temperature synthesis, had porosity that was strongly dependent on the alloy composition. In the case of highly porous alloys, internal oxidation along the pores and grain boundaries was visible. In addition, we were not able to compare the oxidation behavior with binary Fe–Al and Fe–Si alloys prepared in the same way because we were unable to manufacture them in a sufficient quality by the respective method. Despite these problems, we formulated a theory of how the silicon influences the oxidation. Aluminum oxide is the main product of oxidation of Fe–Al-based alloys, as confirmed by numerous research works [5,10,11,12,13]. Therefore, aluminum diffuses to the surface during oxidation and, hence, the material below the oxide layer becomes increasingly depleted by aluminum. When silicon is present in the alloy, the subsurface area depleted by aluminum transforms to silicides, which could be more oxidation resistant than the Al-deficient aluminide and act as a secondary oxidation barrier [9]. It remains a hypothesis since there has not yet been any study on the high-temperature oxidation of iron silicides.

This paper aims to prove our abovementioned hypothesis by comparing the high-temperature oxidation behavior of binary Fe–Al and Fe–Si alloys with FeAl35Si5 and FeAl20Si20 (in wt %) alloys, all of them being prepared by mechanical alloying and spark plasma sintering. The oxidation kinetics and mechanisms of the tested materials are described, compared, and discussed.

## 2. Materials and Methods

The compared alloys of the chemical composition presented in Table 1 were prepared by the combination of mechanical alloying (MA) and subsequent spark plasma sintering (SPS). For mechanical alloying, we used our optimized “ultra-high energy mechanical alloying” process, which was designed in order to produce intermetallics in the shortest possible time [14]. It used the highest energy possible, also employing the friction force and corresponding temperature increase to synthesize intermetallics by thermally activated reactions. Therefore, we used no lubrication during mechanical alloying. In order to prevent oxidation, an argon atmosphere was applied. The process was carried out in planetary ball mill PM 100 CM (Retsch, Haan, Germany) under following conditions—a milling duration of 600 min, change of rotation direction each 30 min, a rotation speed of 400 rpm, powder batch per one milling process 20 g, ball-to-powder weight ratio of 15:1, number of balls as 10, and material of the milling jar and balls made of stainless steel.

For mechanical alloying, the following elemental powders were applied: iron (purity 99.9%, particle size <44 μm, supplied by Strem Chemicals, Newburyport, MA, USA), aluminum (purity 99.7%, particle size <44 μm, supplied by Strem Chemicals, Newburyport, MA, USA), and silicon (purity 99.5%, particle size <44 μm, supplied by Alfa Aesar, Haverhill, MA, USA).

The powder was consolidated by the SPS method by using the HP D10 device (FCT Systeme GmbH, Rauenstein, Germany). The pressure of 48 MPa was applied at 1000 °C for 10 min with a heating rate of 300 °C/min. The cooling rate was set to 50 °C/min in order to prevent the thermal cracking of samples. The weight of the batch for sintering was 5 g. 

The microstructure of the alloys produced by the combination of mechanical alloying and spark plasma sintering was studied by PME3 light microscope (Olympus, Tokyo, Japan) after etching by modified Kroll’s reagent (5 mL HNO_3_, 10 mL HF, and 85 mL H_2_O). Chemical analysis of the individual phases was carried out by the scanning electron microscope (VEGA 3 LMU (TESCAN, Brno, Czech Republic) with EDS analyzer X-max 20 mm^2^ (Oxford Instruments, Abingdon, United Kingdom; SEM–EDS).

Cyclic oxidation tests were carried out at 800 °C in air. The oxidation rate was determined from weight gains caused by the oxide formation on the surface of thermally exposed samples. The duration of one oxidation cycle was 50 h. After each cycle, samples were air-cooled, weighed (analytical balance Pioneer PA224, Ohaus, Parsipanny, NJ, USA, d = 0.0001 g), and heated again to the test temperature. In order to evaluate the oxidation kinetics, the parabolic rate constant was calculated for all oxidation durations according to Equation (1) [15]:

(1) $$k_{p}=\frac{{\left(\frac{\Delta m}{A}\right)}^{2}}{t},$$

where k_p_, Δm, A, and t are parabolic rate constant (g^2^m^−4^s^−1^), weight gain (g), exposed area (m^2^) and duration of oxidation (s), respectively. 

The microstructure of the oxide layers was documented by SEM-EDS. The phase composition of the oxide layers was identified by X-ray diffraction analysis (XRD) using a X’Pert^3^ Powder Pro (PANalytical, Almelo, Netherlands) X-ray diffractometer with Co anode (Kα = 1.789010 × 10^−10^ m) in conventional Bragg–Brentano geometry. Glow discharge optical emission spectroscopy (GDOES) carried out by the means of GD Profiler II (Horiba JobinYvon, Palaiseau, France), was applied for a depth profile chemical analysis of the oxide layers. 

## 3. Results

**Figure 1:**
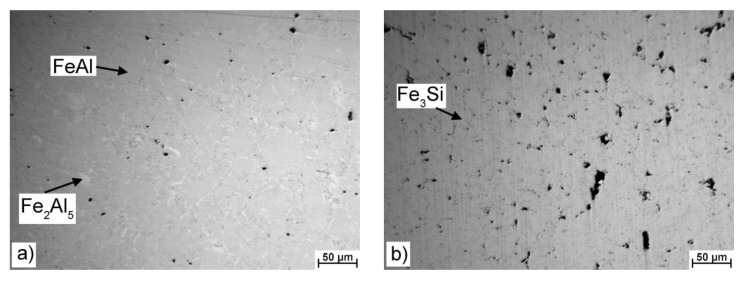

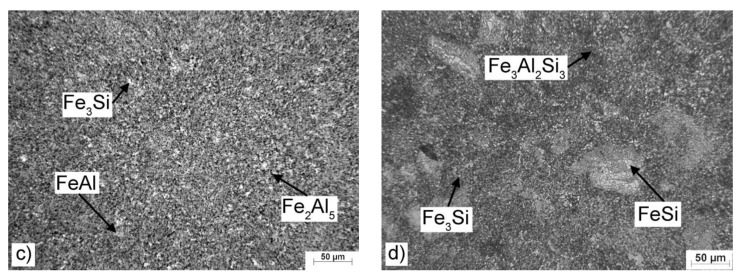
Optical micrographs of the tested materials: (**a**) FeAl32, (**b**) FeSi14, (**c**) FeAl35Si5, (**d**) FeAl20Si20.

The microstructure of the investigated alloys is presented on the optical micrographs in Figure 1. The phases were identified on the basis of the XRD analysis in the previous work [14] and by the EDS chemical microanalysis (see Table 2). It can be seen that the FeAl32 binary alloys contain FeAl (B2 structure) and a minor fraction of Fe_2_Al_5_ phase (Figure 1a). The Fe_2_Al_5_ phase commonly forms during the synthesis of Fe–Al binary alloys by mechanical alloying or self-propagating high-temperature synthesis [16,17]. FeSi14 alloy is composed of iron silicide Fe_3_Si, see Figure 1b. The FeAl35Si5 ternary alloy contains a major amount of FeAl phase, particles of Fe_3_Si, and a minor amount of the Fe_2_Al_5_ phase (Figure 1c). The alloy FeAl20Si20 contains two types of iron silicides (FeSi and Fe_3_Si) in the matrix of the ternary Fe_3_Al_2_Si_3_ phase (Figure 1d). It can be seen in Table 2 that silicon in the iron silicides in ternary alloys is strongly substituted by aluminum. This phenomenon is typical for mechanical alloying, where the composition of the phases could exceed the equilibrium solubility limits due to limited diffusion and different mechanism of the formation of the phases [14]. Distribution of individual phases in the microstructure and their size are almost homogeneous, as can be seen in Figure 1. The porosity of the ternary alloys is almost negligible (below 0.5% of the area in the micrographs), while the porosities of FeAl32 and FeSi14 alloys are approximately 1% and 2%, respectively. It shows poor sinterability of Fe–Si alloys compared with the Fe–Al and Fe–Al–Si alloys. The porosity could affect the oxidation behavior negatively due to an increase in the area of real exposed surface, and in extreme cases, it could cause internal oxidation [9].

**Figure 2:**
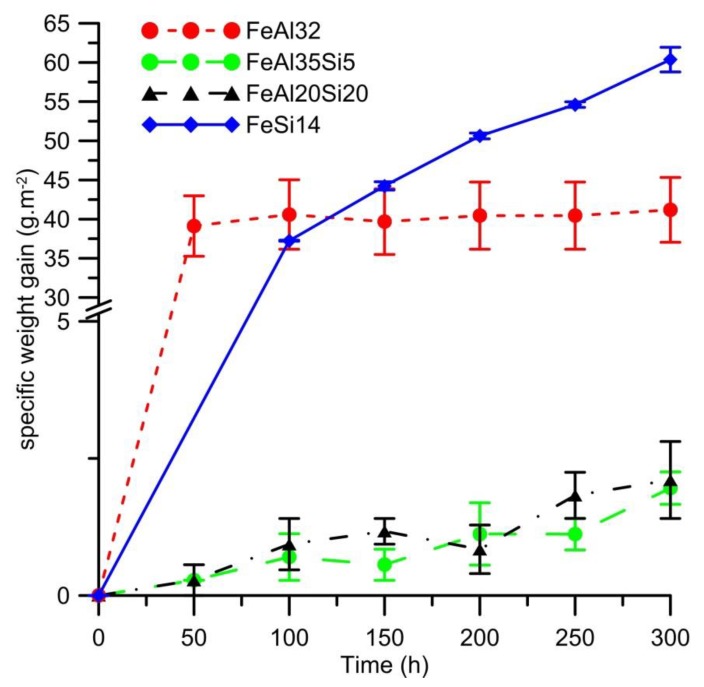
Dependence of specific weight gain (g.m^−2^) on the duration of cyclic oxidation at 800 °C in air.

The oxidation rate of the alloy, represented by weight gains caused by cyclic oxidation, is presented in Figure 2. In the case of the FeSi14 alloy, the shape of the curve is parabolic. It indicates that the oxidation is controlled by the diffusion of oxygen or oxidized element through the oxide layer. In the case of the other alloys, there are some deviations. FeAl32 alloy exhibits a rapid increase in the weight at the beginning of the test and then the weight gains are almost negligible. In cases of the Fe–Al–Si ternary alloys, the weight gains of the samples were very low (0.0002–0.0010 g), just above the measurement error of the balance (0.0001 g). 

After each cycle, the samples were weighed individually, with the crucible in order to be able to evaluate possible spalling of the oxide layer. There were no oxide particles visible in the crucibles after the oxidation test and weight gain measured with and without crucible did not differ more than the measurement error (0.0001 g). Based on this fact, it can be concluded that none of the tested materials tend to experience spalling of the oxide scales during cyclic oxidation test.

**Figure 3:**
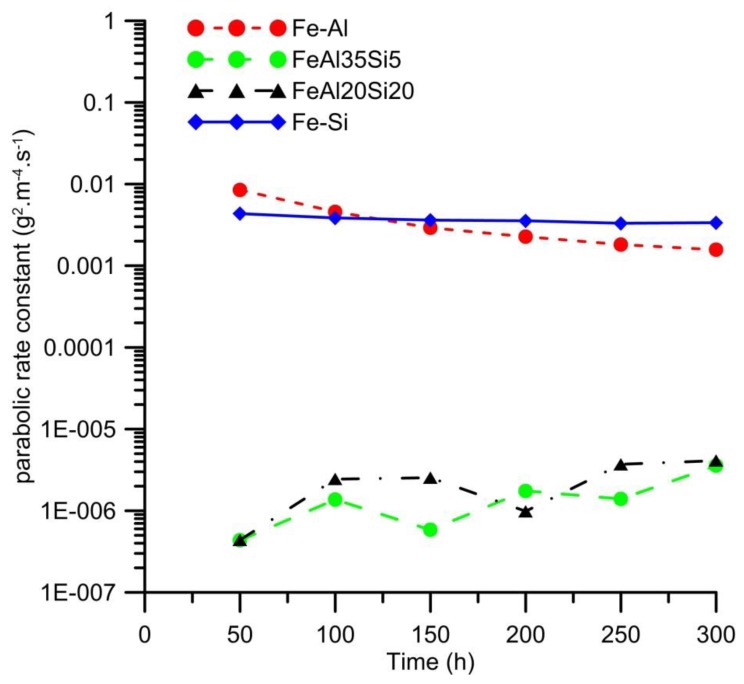
Calculated parabolic rate constants of cyclic oxidation of the tested alloys vs. oxidation duration.

As it has been demonstrated by many studies (e.g., [20,21]) that the oxidation of aluminides usually follows the parabolic law, this behavior was also expected in our case, and hence the parabolic rate constants were calculated for all tested materials and oxidation by the means of Equation (1) for the comparison of the oxidation rate. The results showed that the oxidation of FeSi14 follows the parabolic law right from the beginning of the oxidation process. The other alloys show deviations from the parabolic law, but the values of the calculated parabolic rate constants are still maintained in the same order for all durations of oxidation. In the case of FeAl32 binary alloy, the oxidation rate at the early stage of the oxidation process is higher, then the parabolic rate constant decreases to less than one-half of the value (Figure 3). The probable explanation of this phenomenon is that the oxidation is controlled by the rate of chemical reactions during the early stages. Once the surface is fully covered by a dense oxide layer, the oxidation rate decreases. The oxidation behavior of both ternary Fe–Al–Si alloys is almost the same (Figure 2), exhibiting a three to four orders lower parabolic rate constant than both binary alloys (Figure 3). This shows that the combination of aluminum and silicon provides some kind of synergic effect, leading to extremely low oxidation rates. The variations in the parabolic rate constants of these alloys are most likely caused by the measurement error, because the weight gains by oxidation are extremely low in the case of these samples (0.0002–0.0010 g). 

**Figure 4:**
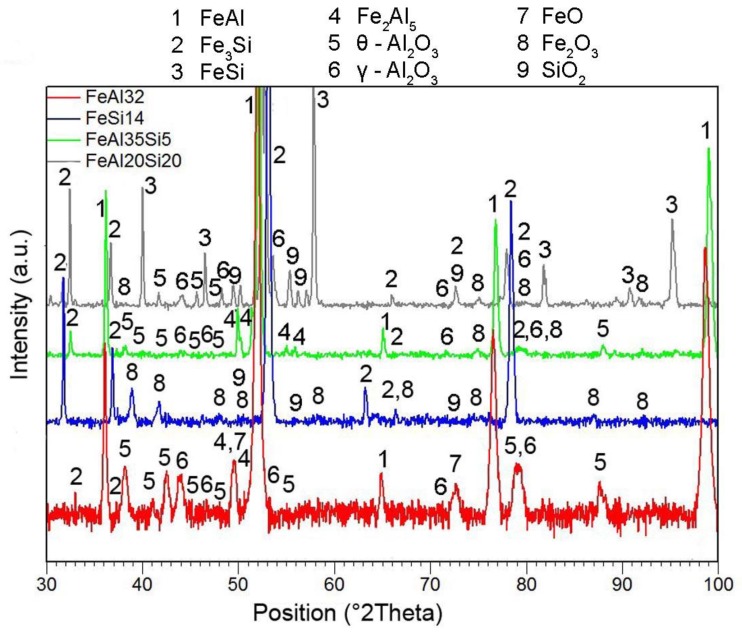
XRD patterns of the oxidized samples.

XRD analysis of the oxidized samples (Figure 4) revealed that the protective layer of aluminum oxide (cubic γ-Al_2_O_3_ and monoclinic θ-Al_2_O_3_) with a minor admixture of FeO is formed on the surface of FeAl binary alloy. On the other hand, Fe_2_O_3_ and SiO_2_ were detected as the oxidation products of Fe–Si alloy by the means of XRD analysis. Both Fe–Al–Si alloys form Al_2_O_3_ (cubic γ-Al_2_O_3_ modification) and Fe_2_O_3_ during the oxidation at 800 °C. In addition, SiO_2_ was also detected on the surface of FeAl20Si20 alloy. In the case of all tested alloys, the components of the basic material (FeAl, Fe_2_Al_5_, Fe_3_Si, and FeSi) were also detected due to the low thickness of the oxide scales. However, the Fe_3_Al_2_Si_3_ ternary phase was not detected, even though it is one of the main constituents of the FeAl20Si20 alloy before oxidation, as seen in Figure 1d. 

**Figure 5:**
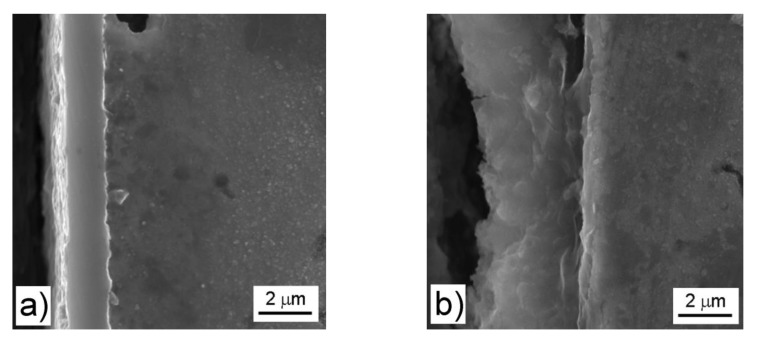

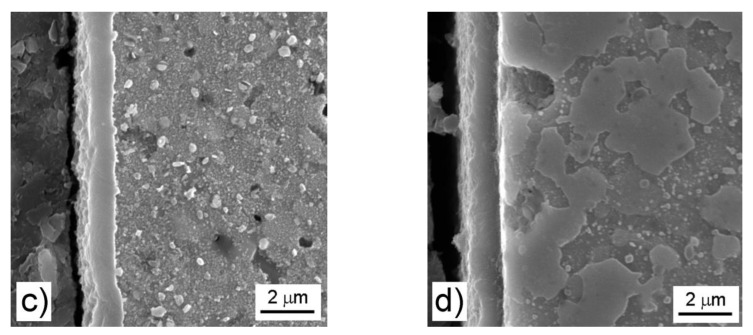
Microstructure of the oxide layers after 300 h of oxidation on (**a**) FeAl32, (**b**) FeSi14, (**c**) FeAl35Si5, (**d**) FeAl20Si20.

The oxide layer on FeAl32 alloy is dense and compact, having the thickness between 2 and 4 µm after the oxidation for 300 h (Figure 5a), depending on the location on the sample. A thicker oxide layer (4–5 µm) can be observed on FeSi14 after the same oxidation duration (Figure 5b). The layer on this material much less uniform than on the FeAl and contains several cracks. On both Fe–Al–Si materials, the oxide layer is thin (between 1 and 2 μm), uniform, and compact without any visible pores or defects (Figure 5c,d). 

The chemical analysis of the oxide layers by EDS (Table 3) revealed that the layers on Fe–Al, FeAl35Si5 and FeAl20Si20 contain mostly aluminum and the Al/O ratio corresponds well to aluminum oxide. In addition, iron was detected in these samples in low amounts (approximately 5–9 wt %). The silicon content in the oxide layers was around 1.4 wt % in FeAl35Si5 and approximately 4.2 wt % in FeAl20Si20 alloys. Compared with the contents of silicon in the alloys, the amounts in the oxide layer are much lower. Hence, it can be concluded that silicon does not participate strongly on the formation of the oxide layers on ternary Fe–Al–Si alloys. On the other hand, the oxide layer on Fe–Si alloy contains an increased proportion of silicon to iron than in the alloy (Table 3).

The EDS chemical analysis was also used in order to describe the changes in chemical composition below the oxide layer. In the case of Fe–Al alloy, strong depletion by aluminum can be seen below the oxide layer (Table 4). In the case of silicon-containing alloy, a slight silicon depletion can be seen. The ternary alloys have a decreasing trend in aluminum content towards the surface as for Fe–Al binary alloy. In addition, the silicon content below the oxide layer is significantly higher than in the core, see Table 4. 

**Figure 6:**
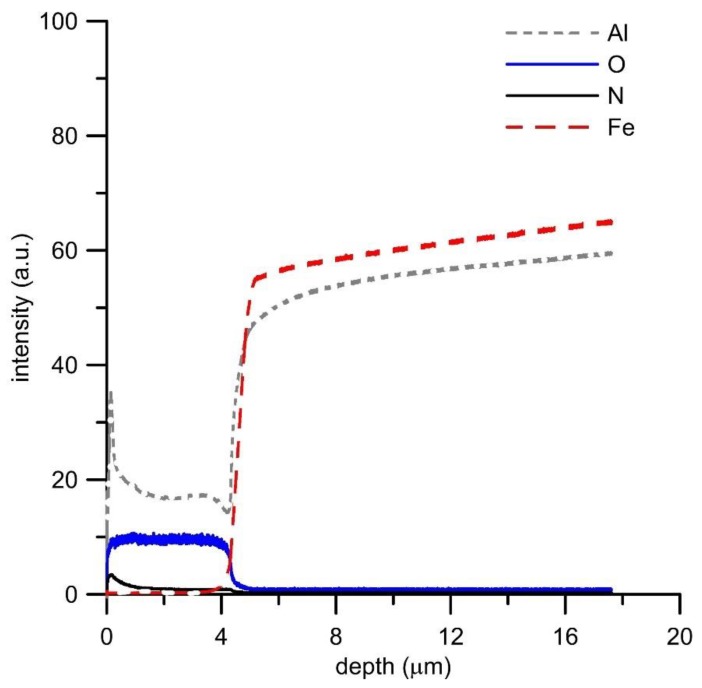
GDOES depth profile of oxidized FeAl32 alloy.

**Figure 7:**
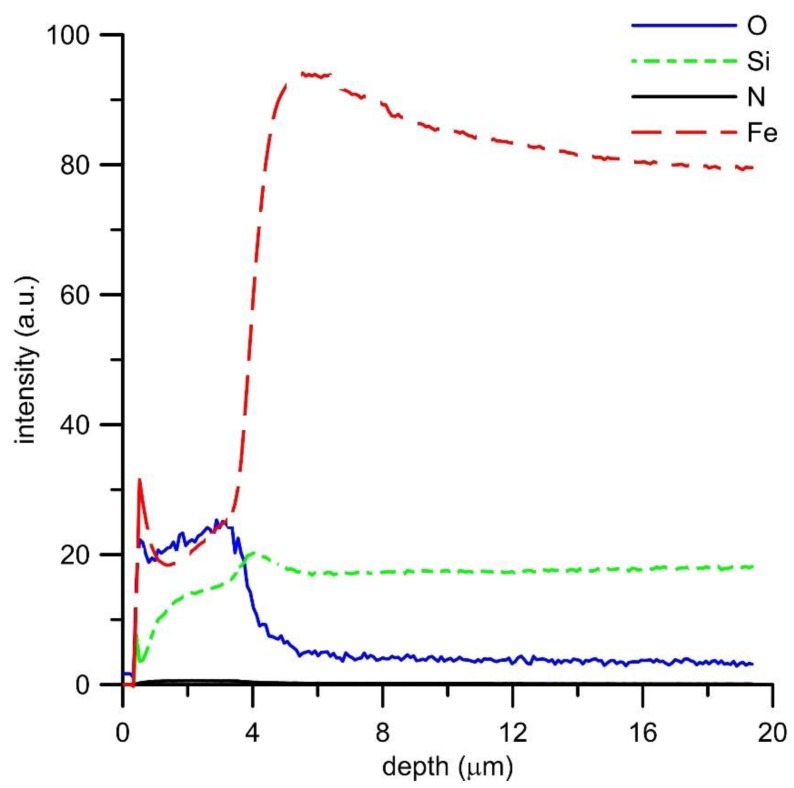
GDOES depth profile of oxidized FeSi14 alloy.

**Figure 8:**
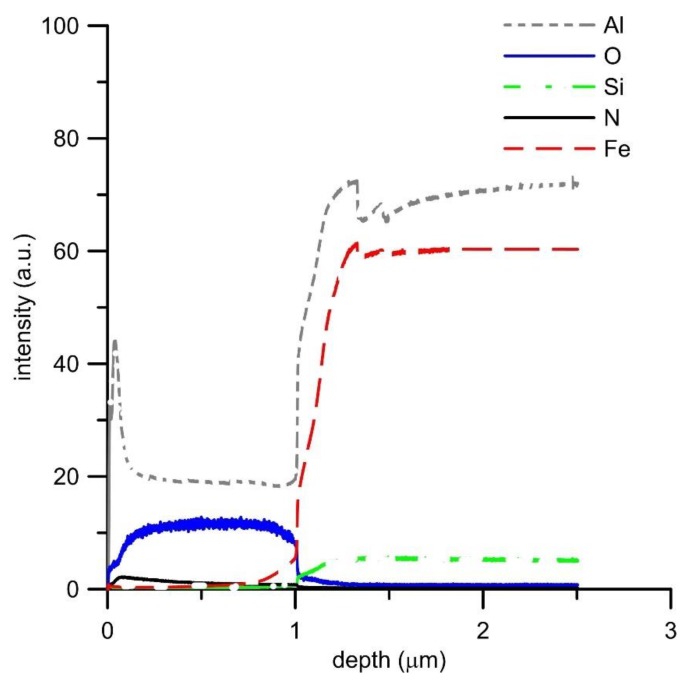
GDOES depth profile of oxidized FeAl35Si5 alloy.

**Figure 9:**
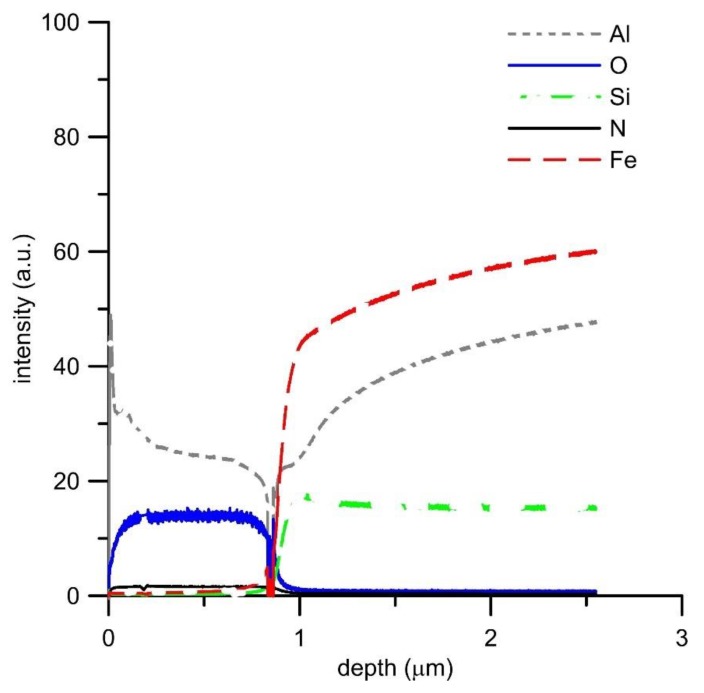
GDOES depth profile of oxidized FeAl20Si20 alloy.

GDOES analysis of the oxide layers (Figure 6, Figure 7, Figure 8 and Figure 9) confirmed the above results, showing that the main oxidation product of FeAl, FeAl35Si5, and FeAl20Si20 alloys is aluminum oxide. The contents of iron and silicon in oxide layers on these samples are very low (Figure 6, Figure 8 and Figure 9). The analysis also proved the presence of nitrogen in the oxide layers, especially on Fe–Al, FeAl35Si5, and FeAl20Si20 alloys. In the case of FeAl20Si20 alloy, there is an observable enrichment of the area below the oxide layer by silicon (Figure 9). In the case of FeAl35Si5 alloy, this phenomenon also exists but is much less significant (Figure 8). On the contrary, this zone is significantly depleted by aluminum, and the depth of this depleted region is the largest in FeAl32 alloy and then it decreases with an increasing amount of silicon in the material. In the case of FeSi14 binary alloy (Figure 6), since silicon concentrates on the inner part of the oxide layer and below it, the material is depleted by silicon.

## 4. Discussion

The presented results showed that the addition of silicon to Fe–Al-based alloys leads to a significant improvement in high-temperature oxidation resistance. A quite surprising fact is that the improvement is almost independent of the ratio between silicon and aluminum. When comparing the oxidation behavior of all tested alloys, we can formulate the following statements.

Fe–Al and Fe–Al–Si alloys tend to form the oxide layer based on aluminum oxide due to a high thermodynamic stability of this oxide (Table 5). At 800 °C, the dominant type of this oxide is a cubic γ-Al_2_O_3_, which is known to have a lower protective effect than α-Al_2_O_3_, which forms at higher temperatures (around 1000 °C) [9]. Despite this fact, the aluminum oxide layer is dense, compact, and has a protective effect slowing down the oxygen diffusion. The content of iron in the oxide layer is low, being bound in FeO or Fe_2_O_3_.

FeSi14 binary alloy forms the oxide layer, which is composed of iron oxide (Fe_2_O_3_) and silicon oxide (SiO_2_) on the inner side of the oxide layer. The presence of both oxides in significant amounts corresponds well with the similar thermodynamic stability of these oxides (Table 5). The fact that silicon accumulates on the inner side of the oxide layer (Figure 7) is probably given by a lower diffusivity of silicon than iron in the oxide layer. The oxidation follows parabolic law, even though the layer is non-uniform and locally cracked. This is probably due to the fact that silicon oxide on the inner side of the oxide layer seals the pores, as detected previously in the case of Ti–Si alloys [22].

The oxidation of Fe–Al and Fe–Al–Si alloys causes depletion of the material below the oxide layer by aluminum. This depletion could cause a decrease of the oxidation resistance, since when the oxide layer becomes damaged, there will not be enough aluminum in the near-surface area to heal the defect. When the alloy contains both silicon and aluminum, silicon concentrates below the oxide layer probably due to its lower diffusivity and lower affinity to oxygen (Table 5). In this zone, which is depleted by aluminum, it reacts with iron to form additional silicides, as was confirmed by the observation (Figure 5d) and conducted analyses. This silicide layer acts as additional oxidation protection, lowering oxygen penetration of the material, and in the case of higher silicon contents, also forming silicon oxide on the inner side of the oxide layer.

For comparison purposes, the thickness of the oxide layers was calculated from the weight gains, assuming that the oxidation product is Al_2_O_3_ in the case of FeAl32 in both Fe–Al–Si alloys, and Fe_2_O_3_ in the case of FeSi14, based on the results of phase and chemical analysis. For the calculation, Equation (2) was applied:

(2) $$d=\;\frac{\Delta m\cdot x\cdot M\left(oxide\right)}{A\cdot \rho \cdot x\cdot M\left(O\right)}\cdot 10000,$$

where d is the layer thickness (μm), x is the number of oxygen atoms in the formula of oxide (3 for both aluminum oxide and iron oxide), M(O) is the molar weight of oxygen (16 g.mol^−1^), A is the area of exposed surface (cm^2^), ρ is the density of oxide (3.95 g.cm^−3^ for Al_2_O_3_ and 5.24 g.cm^−3^ for Fe_2_O_3_), and M(oxide) is the molar weight of oxide (102 g.mol^−1^ for Al_2_O_3_ and 160 g.mol^−1^ for Fe_2_O_3_). The results of the calculation are presented in Table 6. There is a very good agreement between the values measured on SEM micrographs and calculated ones in the case of the ternary Fe–Al–Si alloys. On the other hand, the binary alloys show a big misfit between calculated and observed layer thickness. The probable reason is the internal oxidation and oxygen uptake to the binary alloys. This is confirmed in Figure 5, where the changes in microstructure and the pores below the oxide layer are visible in FeAl32 and FeSi14 alloys. On the other hand, in the case of ternary alloys, only an increased amount of oxidation-resistant silicides had been detected below the oxide layer without any trace of internal oxidation. 

## 5. Conclusions

In this paper, the oxidation behavior of Fe–Al, Fe–Si and Fe–Al–Si alloys prepared by mechanical alloying and spark plasma sintering was studied and compared with the following main results:

- Fe–Al and Fe–Al–Si alloys form the oxide layer based on aluminum oxide with minor amounts of silicon and aluminum.
- Fe–Si alloy oxidizes forming the mixture of iron oxide and silicon oxide.
- The oxidation rate of Fe–Al–Si alloys is three to four orders lower than that of Fe–Al and Fe–Si alloys due to the presence of dense aluminum oxide layer and a layer of iron silicides below it, which act as a secondary oxidation protection.

## Figures and Tables

**Table 1:** Nominal chemical composition of the tested alloys.

	Weight %			Atomic %		
	Fe	Al	Si	Fe	Al	Si
**FeAl32**	68	32	0	50.7	49.3	
**FeSi14**	86	0	14	75.5		24.5
**FeAl35Si5**	60	35	5	42.2	50.8	7.0
**FeAl20Si20**	60	20	20	42.5	29.3	28.2

**Table 2:** Average chemical composition (analyzed by SEM–EDS) and crystal structures of the phases identified in tested alloys.

Studied Alloy	Identified Phase	Chemical Composition (atom %)			Crystal Data (from Ref. [18,19])		Ref.
		Fe	Al	Si	Crystal Structure	Space Group	
**FeAl32**	**FeAl**	52.2 ± 1.2	47.8 ± 1.2	-	B2	$Pm\bar{3}m$	[18]
	Fe_2_Al_5_	27.3 ± 0.5	72.7 ± 0.5	-	-	Cmcm	[18]
**FeSi14**	Fe_3_Si	75.5 ± 0.3	-	24.5 ± 0.3	D0_3_	$Fm\bar{3}m$	[18]
**FeAl35Si5**	FeAl	57.9 ± 1.3	40.1 ± 1.2	1.0 ± 0.1	B2	$Pm\bar{3}m$	[18]
	Fe_3_Si	75.6 ± 0.3	6.9 ± 1.0	17.5 ± 0.7	D0_3_	$Fm\bar{3}m$	[18]
	Fe_2_Al_5_	27.1 ± 0.3	72.1 ± 0.5	0.8 ± 0.5	-	Cmcm	[18]
**FeAl20Si20**	Fe_3_Si	72.0 ± 0.6	12.8 ± 0.6	15.2 ± 0.5	D0_3_	$Fm\bar{3}m$	[18]
	FeSi	48.6 ± 1.4	9.8 ± 1.2	42.6 ± 1.4	B20	P2_1_3	[18]
	Fe_3_Al_2_Si_3_	37.6 ± 0.3	24.9 ± 0.2	37.4 ± 0.3	-	P-1	[19]

**Table 3:** Chemical composition of the oxide layers after 300 h of oxidation at 800 °C (in wt %, EDS).

	Weight %				Atomic %			
	Fe	Al	Si	O	Fe	Al	Si	O
**FeAl32**	5.0 ± 1.1	49.1 ± 0.6	-	45.9 ± 0.2	1.9 ± 0.6	38.1 ± 0.6	-	60.0 ± 0.4
**FeSi14**	56.7 ± 1.5	-	14.6 ± 0.7	28.7 ± 0.9	30.5 ± 0.7	-	15.7 ± 0.7	53.8 ± 1.4
**FeAl35Si5**	7.5 ± 0.9	44.6 ± 1.1	1.4 ± 0.3	46.5 ± 0.7	2.8 ± 0.5	34.8 ± 1.0	1.1 ± 0.3	61.3 ± 1.3
**FeAl20Si20**	8.2 ± 0.8	41.3 ± 1.3	4.2 ± 0.6	46.3 ± 0.7	3.1± 0.4	32.4 ± 1.1	3.2 ± 0.6	61.3 ± 1.3

**Table 4:** Chemical composition of the material below the oxide layer after 300 h of oxidation at 800 °C (in wt %, EDS).

	Depth Below Oxide Layer (µm)	Fe	Al	Si
**FeAl32**	10	73.9 ± 0.7	26.1 ± 0.7	-
	30	71.7 ± 0.7	28.3 ± 0.7	-
	50	69.3 ± 0.6	30.7 ± 0.6	-
**FeSi14**	10	86.8 ± 0.6	-	13.2 ± 0.6
	30	86.3 ± 0.7	-	13.7 ± 0.7
	50	86.0 ± 0.6	-	14.0 ± 0.6
**FeAl35Si5**	10	61.3 ± 0.5	32.8 ± 0.5	5.9 ± 0.5
	30	62.5 ± 0.5	33.4 ± 0.5	4.2 ± 0.5
	50	61.8 ± 0.5	33.4 ± 0.5	4.8 ± 0.5
**FeAl20Si20**	10	60.0 ± 0.7	16.4 ± 0.7	23.6 ± 0.7
	30	60.4 ± 0.7	18.6 ± 0.7	21.0 ± 0.7
	50	59.3 ± 0.6	20.3 ± 0.6	20.4 ± 0.6

**Table 5:** Gibbs energy (ΔG_f_) of formation of oxides at 800 °C [23].

Oxide	ΔGf 800 °C (kJ∙mol^−1^)
**Al_2_O_3_**	−1778
**Fe_2_O_3_**	−982
**SiO_2_**	−983

**Table 6:** Oxide layer thickness calculated from weight gains after 300 h of oxidation and measured on SEM micrographs.

Alloy	Calculated Oxide Thickness (μm)	Measured Oxide Thickness (μm)
**FeAl32**	22.0 ± 3.0	2–4
**FeSi14**	38.0 ± 1.0	4–5
**FeAl35Si5**	1.3 ± 0.3	1–2
**FeAl20Si20**	1.4 ± 0.5	1–2
